# Guide to Understanding Drosophila Models of Neurodegenerative Diseases

**DOI:** 10.1371/journal.pbio.0060053

**Published:** 2008-02-26

**Authors:** George R Jackson

## Abstract

Demystifying how genetic studies in*Drosophila* inform human disease conditions, this article highlights two studies that identify genetic modifiers of neurodegeneration.

In the decade since human genes associated with neurodegenerative disease were first used in flies to create pathological phenotypes [1–5], a minor industry has sprung up using flies as a model to study the mechanisms underlying central nervous system malfunction in humans. Why study neurodegeneration in flies? Their small size, rapid generation time, and low costs for maintenance as compared to mammalian models make them attractive enough. The true value of flies to the study of neurodegenerative disorders, however, is their capacity to provide a platform for unbiased genetic screens to identify components of pathological pathways. If expression of pathological human genes in the fly successfully generates an abnormal phenotype, such as slowed motor activity or degeneration of the retina, this phenotype can then be used in conjunction with the rich genetic toolbox that Drosophila researchers have developed over the last 90 years to identify pathways that contribute to this degeneration. This approach is unbiased, i.e., it does not depend upon prior assumptions about mechanisms underlying disease, and genome-wide screens can be carried out in the fly that would be difficult if not impossible to carry out using mouse models.

Key to such an approach is how similar flies are to humans. A stunning 75% (approximately) of the genes implicated in human genetic disorders have at least one homolog in the fruit fly (see http://superfly.ucsd.edu/homophila/ for further information). In general, fundamental aspects of cell biology relevant to processes as diverse as cell cycle regulation, synaptogenesis, membrane trafficking, and cell death are similar in Drosophila and humans. Of course, there are important differences between flies and humans; for example, the circulatory system is much simpler in the fly, and cognitive processes are much less complex. Nonetheless, the fly has proved itself as a useful adjunct to mammalian models for neurodegenerative diseases.

A variety of neurodegenerative disorders have been modeled in the fly (for reviews, see [6–10]); perhaps the best established and most robust models are those associated with a group of inherited disorders that are all caused by the same mechanism: expansion of an unstable CAG repeat resulting in expression of proteins containing expanded polyglutamine tracts. The best known of these disorders is Huntington disease, but there are a number of somewhat similar disorders also caused by a CAG repeat expansion that are collectively referred to as the spinocerebellar ataxias (SCAs). These are generally adult-onset, progressive neurodegenerative disorders that feature impaired coordination due to degeneration of the cerebellum [11]. The different SCAs may have slightly different symptoms in addition to impaired coordination, such as tremor in SCA type 2 (SCA2) similar to that seen in Parkinson disease or muscular atrophy due to nerve damage in SCA3, but all are inexorably progressive. Some symptoms, such as impaired swallowing or gait ataxias, may be mildly improved by medications, assistive devices, or physical therapy, but no disease-modifying treatments exist. The proteins in which the polyglutamine expansions occur in each disorder show no obvious similarities and are referred to as ataxins 1, 2, 3, etc. (Atx1, Atx2, Atx3, etc.).

Although the syndromic classifications of neurodegenerative disorders that began to be developed in the nineteenth century focused on distinctions between different disorders, more recent pathological and molecular analyses have begun to identify commonalities between what have traditionally been thought of as distinct diseases, as well as crosstalk between seemingly unrelated disease-associated proteins. In this issue of *PLoS Biology*, Derek Lessing and Nancy Bonini describe an interaction in which Atx2 contributes to the pathogenicity of Atx3 [12]. This report comes on the heels of similar work by Juan Botas and colleagues describing an interaction between Atx2 and Atx1 [13]. Here, I set out to demystify the creation and deployment of fly models of neurodegenerative diseases, and to put the current studies of interaction among ataxins in perspective.

## How and Why Are Human Disease-Associated Genes Expressed in the Fly?

**Figure 1:**
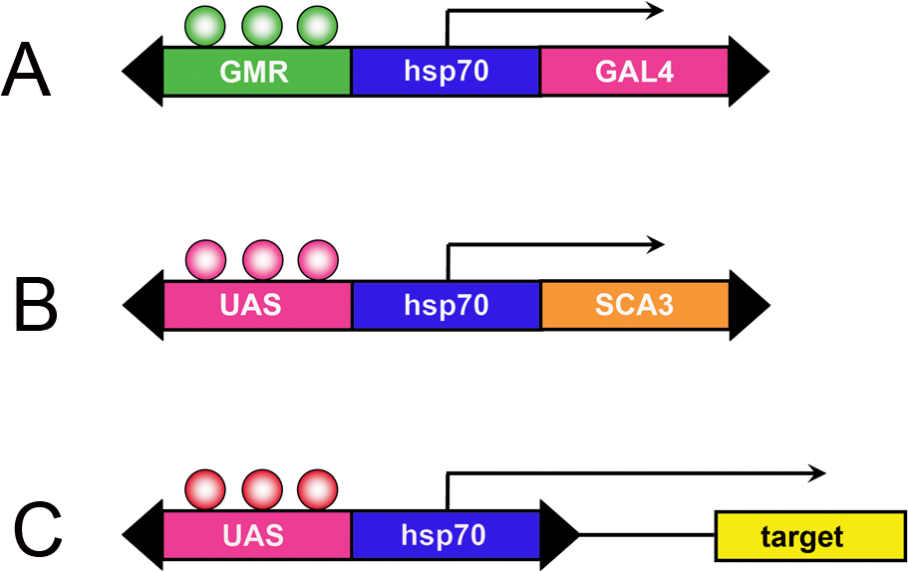
Schema for Generation of Fly Models of Neurodegenerative Diseases A variety of driver lines are available that express the yeast transcriptional co-activator GAL4; shown is GMR-GAL4, which uses regulatory DNA that responds to the *glass* transcription factor to induce expression of GAL4 in all cells of the eye beginning in larvae (A). The cDNAs encoding human disease-associated genes, such as that for Atx3, which causes SCA3, are subcloned into the UAS expression construct (B) and used to generate transgenic flies. When these flies (B) are crossed with those of a driver line (A), GAL4 is expressed in the progeny and induces expression of the disease gene, producing an abnormal eye phenotype that can readily be scored under the dissection microscope. A stable tester stock can be established that expresses both of these transgenes and used to carry out genetic screens. In the case of the EP element (C), in the presence of GMR-GAL4 (A), genomic sequences near the EP element can be identified that modulate severity of the tester stock.

The most common means of expressing human neurodegenerative genes in Drosophila makes use of the binary GAL4-dependent upstream activating sequence (GAL4/UAS) system [14] (Figure 1). The gene of interest is subcloned into the UAS expression construct (Figure 1B), which is microinjected into fly embryos to establish transgenic lines. Once these lines are in hand, disease genes can be expressed in a variety of tissues using available stocks that express the yeast transcriptional co-activator GAL4. One of the most useful of these driver lines [15] expresses GAL4 in all cells of the eye under control of the *glass* transcription factor (Figure 1A). The fly eye is particularly useful in the context of genetic screens for genes that affect tissue integrity, as fertility is retained even in the presence of severe retinal degeneration. So in order to achieve expression of a human SCA gene, one can simply take the new flies that have been generated containing the human cDNA (Figure 1B) and cross them with an already established strain of flies that express GAL4 in the retina (Figure 1A). Both transgenes will be expressed in the progeny of these flies (Figure 1A and Figure 1B); thus when GAL4 protein is expressed under control of the endogenous *glass* promoter, it will activate expression of the SCA transgene.

**Figure 2:**
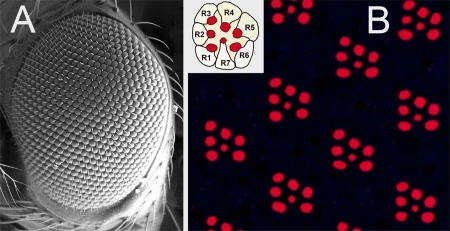
Structure of the Fly Eye (A) Scanning electron micrograph of the wild-type fly eye shows hundreds of ommatidia. (B) Internal view of rhabdomeres within ommatidia. Single confocal optical section of whole mount retina stained with phalloidin-TRITC (tetramethyl rhodamine isothiocyanate). Apical (toward the surface of the eye) tangential sections identify rhabdomeres of seven photoreceptor neurons. Virtually identical images can be obtained using the optical neutralization technique (“pseudopupil”) and used to quantitate effects of genetic modifiers of neurodegeneration.

Genetic screens using the fly eye have been enormously successful in the study of neurodegeneration. The compound eye of Drosophila melanogaster is composed of some 800 repeating subunits, called ommatidia (Figure 2A). Each ommatidium contains eight photoreceptor neurons, seven of which are visible in tangential sections taken toward the external surface of the eye (Figure 2B). Each photoreceptor elaborates a membranous organelle, called a rhabdomere, which is involved in phototransduction. In Figure 2B, actin in the rhabdomeres has been stained with fluorescently labeled phalloidin. Degeneration of photoreceptor neurons can be quantitated by counting the rhabdomeres in each ommatidium, using an optical neutralization technique (often referred to as the “pseudopupil”) to obtain images similar to that in Figure 2B. This technique is rapid, reproducible, does not require staining using antibodies or dyes, and has been used with great success since it was first introduced in studies of human neurodegenerative diseases almost ten years ago [2].

## How Are Fly Models Used in Unbiased Genetic Screens?

Once a UAS line encoding a pathogenic gene (e.g., expanded Atx3; Figure 1B) has been established, it can be stably placed in a combination with a driver line such as *glass* multimer reporter (GMR)-GAL4 (Figure 1A). This stabilized stock is now ready for use in genetic screens that employ transposon or deletion libraries. One of the most commonly used transposon libraries utilizes the enhancer–promoter (EP) element (Figure 1C), which is essentially an “empty” UAS construct that drives expression of nearby genomic sequences in the presence of GAL4 [16]. What this means in practical terms is that a large number of crosses can be set up with a tester strain that contains the driver line (“A”) and the UAS line (“B”). These flies are then crossed with a large number of individually derived stocks that contain insertions of this EP in different regions of the genome. The progeny of these crosses will then contain three different transgenes: the GAL4 driver (A), the UAS lines (B), and an EP insertion. In these crosses, the GAL4 will not only drive expression of the SCA gene, but will also drive expression of endogenous fly genes in the vicinity of the EP insertion. In a subset of these crosses (A + B + C), the eye phenotype may be either more or less severe than in the control cross (A + B only); one can then infer that the target gene in the vicinity of the EP insertion in C is a player in the pathological process set in motion by expression of the pathological SCA gene.

## A Tale of Two Ataxins

One of the genetic modifiers identified in the work described by Lessing and Bonini in this issue corresponded to the endogenous fly version of Atx2 (dAtx2): the rough eye phenotype produced by mutant human Atx3 was stronger in the presence of gain-of-function dAtx2 alleles. Leo Pallanck and colleagues had previously characterized this gene [17], and the enhancement of the Atx3 phenotype was confirmed using directed overexpression of dAtx2 (i.e., UAS-dAtx).

Retinal phenotypes are certainly useful for identifying genetic modifiers of disease; however, retinal degeneration is not a prominent feature of the SCAs (with the notable exceptions of SCA2 [18] and SCA7 [19]). Nonetheless, because the retina can easily be viewed by dissecting microscope and can be dramatically disrupted without compromising the overall health of the fly, eye phenotypes are propitious for genetic screens. Of course, one would like to see new genes identified with the retina to be studied further in other tissues that may be more characteristically affected in neurodegenerative disorders, such as brain or muscle. Overexpression of dAtx2 alone using the GMR-GAL4 driver produced abnormal eye phenotypes [17]. Given that the SCAs are adult-onset disorders, there is understandable bias in the field against models that rely exclusively on developmental phenotypes. In addition, although the mechanisms underlying adult-onset cell death may be quite similar to those that take place in developmental apoptosis in some instances, this may not hold true in others. For these reasons, the authors turned to a later-onset retinal driver, rhodopsin-1 [20]. This driver generates expression shortly before emergence of the adult fly in the outer photoreceptors (R1–R6 in Figure 2B), and has been used successfully in prior studies of polyglutamine modifiers [21]. Quantitative analysis of images obtained using optical neutralization confirmed the genetic interaction of overexpressed Atx3 and dAtx2; conversely, loss-of-function alleles of dAtx2 suppressed mutant Atx3-induced phenotypes.

Modifier validation is often carried out in other neuronal populations in addition to the retina. This can be challenging to carry out in the central nervous system due to technical difficulties with quantitation using pan-neuronal drivers. Lessing and Bonini turned to sensory neurons in the wing margin and made clever use of the MARCM (mosaic analysis with a repressible cell marker) [22] technique to generate clones that were mutant for two copies of dAtx2. Using this technique, fluorescent Atx3-expressing neurons were quantitated in living flies; these again highlighted a dosage-sensitive role of dAtx2 in contributing to neurodegeneration caused by mutant Atx3.

Given that a variety of lines expressing human neurodegenerative disease genes are available, one simple means of assessing the specificity of a genetic interaction is by comparing modifier effects in the context of other proteins. This was of particular interest in the case of the Atx2/Atx3 interaction, given that Atx2 had previously been identified as a modifier of Atx1. Enhancement of the polyglutamine phenotypes also was observed with an exon-1 Huntingtin construct [23], but not a larger fragment [24], indicating some importance of protein context in the interaction. These data are complemented by those reported by Botas and colleagues, who used a mutant Huntingtin construct intermediate in length between the two used by Bonini and Lessing; this Huntingtin phenotype also was not modified by dAtx2.

Human Atx2 is unusual among the ataxins in that it is almost exclusively a cytoplasmic protein, although it has been identified in nuclear inclusions in SCA3 post-mortem brain [25]. Immunohistochemical and western blot data provided by Lessing and Bonini suggested a role for Atx2 in regulating aggregation of mutant Atx3, and a construct lacking the PAM2 motif (PABP-interacting motif 2) of Atx2 was incapable of providing enhancement. Endogenous dAtx2 was recruited into inclusions. This finding mirrors that of Botas and colleagues in *PLoS Genetics*, who in addition showed that forced nuclear expression of dAtx2 enhanced its toxicity [13]. The PAM2 motif of Atx2 mediates its interaction with poly(A)-binding protein (PABP), and the Atx3 phenotype was sensitive to PABP dosage.

## Quo Vadis, Ataxia?

This work is basic research; thus neither the work of Lessing and Bonini nor that recently reported by Botas and colleagues, unfortunately, has identified a magic bullet for treatment of inherited ataxias or related polyglutamine disorders. They do suggest however that important interactions may exist between disease-associated proteins that have traditionally been associated strictly with single diseases. Importantly, this crosstalk suggests that a cure for one may be a cure for all. Analogous to the crosstalk that may take place between pathogenic fragments of amyloid precursor protein and tau in the context of Alzheimer disease [26], further study of interactions between ataxins may provide important mechanistic insights into disease pathogenesis. If Atx2 does indeed play a role in amplifying toxicity of both Atx1 and Atx3, then perhaps dampening of this activity by genetic or pharmacological means could lead to a disease-modifying as opposed to a purely symptomatic treatment.

Could effecting small changes in the toxicity of mutant Atx3 by genetic or pharmacological means make a difference in the lives of patients? Age of onset for polyglutamine repeat disorders is not determined exclusively by length of the repeat expansion, and both genetic and environmental factors are likely to play a role in disease onset and progression. Even minor decreases in mutant protein toxicity might be translated as major improvements in quality of life for those afflicted with these incurable disorders.

Thus the simple fruit fly has been used to rapidly and readily screen through a large number of potential interacting genes to uncover a surprising and potentially therapeutically important interaction between two genes that previously had not been known to interact or share common features, apart from a polyglutamine tract. The beauty of this sort of work is that even a small group with limited resources can carry it out using fly models. The next step, obviously, will be to further analyze this interaction using mammalian models. Will a mouse that expresses mutant Atx3 develop more severe neurodegeneration when it is crossed with one that overexpresses normal Atx2? These and similar experiments will be key steps in further investigating the significance of the findings described by Lessing and Bonini. Given that previous insights into neurodegeneration learned from Drosophila, such as chaperone and histone deacetylase inhibitor rescue [6–9], have translated to mammalian models, this tale of two ataxins provides a launching ground to take these new findings from fly to mouse and ultimately to human patients.

## Abbreviations

EP: enhancer–promoter
GAL4/UAS: GAL4-dependent upstream activating sequence
GMR: *glass* multimer reporter
PABP: poly(A)-binding protein
PAM2 motif: PABP-interacting motif 2
SCA: spinocerebellar ataxia
